# CXCL13 promotes thermogenesis in mice via recruitment of M2 macrophage and inhibition of inflammation in brown adipose tissue

**DOI:** 10.3389/fimmu.2023.1253766

**Published:** 2023-10-23

**Authors:** Lijun Xie, Huiying Wang, Dan Wu, Feng Zhang, Wei Chen, Yuqing Ye, Fang Hu

**Affiliations:** National Clinical Research Center for Metabolic Diseases, Key Laboratory of Diabetes Immunology, Ministry of Education, Department of Metabolism and Endocrinology, the Second Xiangya Hospital of Central South University, Changsha, Hunan, China

**Keywords:** CXCL13, BAT, thermogenesis, M2 macrophages, TNFα

## Abstract

**Introduction:**

Brown adipose tissue (BAT) is mainly responsible for mammalian non-shivering thermogenesis and promotes energy expenditure. Meanwhile, similar to white adipose tissue (WAT), BAT also secretes a variety of adipokines to regulate metabolism through paracrine, autocrine, or endocrine ways. The chemokine C-X-C motif chemokine ligand-13 (CXCL13), a canonical B cell chemokine, functions in inflammation and tumor-related diseases. However, the role of CXCL13 in the adipose tissues is unclear.

**Methods:**

The expression of CXCL13 in BAT and subcutaneous white adipose tissue (SWAT) of mice under cold stimulation were detected. Local injection of CXCL13 into BAT of normal-diet and high-fat-diet induced obese mice was used to detect thermogenesis and determine cold tolerance. The brown adipocytes were treated with CXCL13 alone or in the presence of macrophages to determine the effects of CXCL13 on thermogenic and inflammation related genes expression in vitro.

**Results:**

In this study, we discovered that the expression of CXCL13 in the stromal cells of brown adipose tissue significantly elevated under cold stimulation. Overexpression of CXCL13 in the BAT via local injection could increase energy expenditure and promote thermogenesis in obese mice. Mechanically, CXCL13 could promote thermogenesis via recruiting M2 macrophages in the BAT and, in the meantime, inhibiting pro-inflammatory factor TNFα level.

**Discussion:**

This study revealed the novel role of adipose chemokine CXCL13 in the regulation of BAT activity and thermogenesis.

## 1 Introduction

Worldwide prevalence of obesity has become a major public health problem, which significantly increases the risk of type 2 diabetes, fatty liver diseases, hypertension, myocardial infarction, stroke, many types of cancer, and COVID-19, leading to a decrease in life quality and expectancy (1–3).

The occurrence of obesity is closely related to the excessive accumulation of adipose tissue and the imbalance of energy intake and expenditure. BAT is the major tissue of non-shivering thermogenesis in mammalians, while WAT is the main storehouse of lipids in the form of triglycerides (TG) (4). Many studies have shown the beneficial effects of BAT activation on obesity, insulin resistance, and hyperlipidemia in mice. However, because the amount of metabolically active BAT is particularly decreased in individuals with obesity or diabetes, new strategies are needed to increase adaptive thermogenesis and energy expenditure (5, 6).

Recent studies have suggested that BAT can be considered as an endocrine organ. BAT-derived cytokines or adipokines, also called batokines, act in a paracrine or autocrine manner to promote BAT thermogenesis in the way of hypertrophy, hyperplasia, angiogenesis, innervation, and blood flow (7). Therefore, understanding the role of adipokines or other molecules in the BAT will help us find a new target for the treatment of obesity and related diseases. Immune cells are key functional regulators of BAT and WAT. Under cold conditions, BAT establishes a complex paracrine network of cytokines, including IL4, IL13, IL5, and IL33 to activate immune cells, including M2 macrophages, eosinophils, and type 2 innate-like lymphocytes (7). Besides cytokines, chemokines secreted by brown adipocytes, adipose precursor cells, or immune cells interact with cytokines to regulate BAT functions.

CXCL13 (C-X-C motif chemokine ligand 13) is a member of the CXC chemokine family and a canonical B cell-attracting chemokine. CXCL13 is mainly produced by stromal cells in secondary lymphoid organs (including the spleen, lymph nodes, tonsils, and Peyer’s nodes), and its function is mediated by its specific receptor CXCR5 (8). Under normal conditions, CXCL13 binds to CXCR5 to exert its biological effects and participates in the homing of circulating naive B lymphocytes to lymph nodes (9). In addition to the regulation of B lymphocytes, CXCL13 has been reported to be involved in the M2 macrophage polarization and tumor progression in myeloma osteolytic (10). Most of these studies investigated the effects of CXCL13 secreted from CD68+CD206+ macrophages on tumor cells (11, 12), however, whether the CXCL13 could regulate macrophages remains to be explored.

Previous research has shown that the expression of CXCL13 is upregulated during adipogenesis. However, treating precursor adipocytes with CXCL13 does not affect adipogenesis (13). The expression of CXCL13 and its receptor CXCR5 are elevated in both subcutaneous white adipose tissue (SWAT) and visceral adipose tissue (VAT) under hypoxic conditions and in individuals with obesity (14). Besides, in pericardial adipose tissues (PATs), CXCL13 and IL-33 can recruit IL-10-producing CD5+ B cells to alleviate myocardial injury in acute myocardial infarction (15). In a 3D paracrine system for wound healing treatment of macrophages and adipose precursor cells, *Cxcl13* gene expression is positively related to the expression of M2 macrophage genes including *Arg1* and *Cd206* (16).

Previous research about the function of CXCL13 is focused on the immune cells, while the role of CXCL13 in adipose tissues is still uncertain. Our preliminary study has found that the CXCL13 level was significantly elevated in the BAT under cold stimulation, while the action of CXCL13 in adipose tissue thermogenesis is unclear. In this study, we have found that CXCL13 could reduce inflammation and promote the thermogenesis of BAT through M2 macrophage polarization.

## 2 Materials and methods

### 2.1 Reagents

Recombinant Murine BCA-1/BLC (CXCL13; 250-24), recombinant murine IL-4 (214–14), and recombinant murine M-CSF (315–02) were purchased from PeproTech Inc. (NJ, USA). Lipopolysaccharides (LPS, L2654), anti-β-actin (A3854), and anti-UCP1 (U6382) primary antibodies were obtained from Millipore Sigma Inc. (USA). Mouse anti-PRDM16 antibody (ab303534) was purchased from Abcam Inc. (USA). Mouse anti-CXCL13/BLC/BCA-1 antibody (AF470) and CXCL13 ELISA Kits (MCX130) were purchased from RDSystems Inc. (USA). Mouse adipose triglycerides lipase (ATGL, #2138), Acetyl-CoA Carboxylase (ACC, #3676), Phospho-Acetyl-CoA Carboxylase (Ser79, *p*-ACC, #3661), hormone-sensitive lipase (HSL, #4107) and Phospho-HSL (Ser565, *p*-HSL, #4137) were purchased from Cell Signaling Technology Inc. (USA). Anti-mouse TNFα primary antibody (A11534) was purchased from ABclonal Technology Co., Ltd. (China). Mouse TNFα ELISA Kits (CME0004) were purchased from 4A Biotech., Ltd (China). Flow cytometric antibodies including PE/cy7-F4/80 (#123114), APC-CD19 (#115512), PE-CD206 (#141705), FITC-CD45 (#103108), Pacific Blue-CD11b (#101224), PerCP/cy5.5-CD3 (#100328) and PerCP-CD11c (#117325) were purchased from Biolegend Inc. (USA).

### 2.2 Animal studies

Male C57BL/6J mice were purchased from Slac Laboratory Animal Inc. (Shanghai, China) and fed with a high-fat diet (HFD, 60 Kcal% fat, Cat #D12492; Research Diets Inc., NJ, USA) for 8 weeks. All mice were housed in a temperature-controlled environment (22°C) with a 12:12h light/dark cycle and free access to food and water.

For the cold tolerance test, 8-week-old normal diet (ND) C57BL/6J mice (cold 1 day vs. cold 7 days) and 8-week-HFD mice (CXCL13 group vs. PBS group) were kept in a single cage with sufficient water and Ad libitum, and the cages were set at 6°C or room temperature (22°C). The rectal temperature of mice was measured with a temperature sensor (TH212, Zhonglutong Inc., China).

The rodent’s surface temperature was measured using an infrared digital thermal imaging camera (TiX620, Fluke, USA). Infrared pictures of non-anesthetized animals were taken and analyzed using the SmartView Classic 4.4 software (Fluke). Average temperature values from the interscapular and the dorso-lumbar areas were retrieved, and a ratio of the two was calculated as a measurement of interest.

For CXCL13 injection, ND and HFD-fed mice were anesthetized with isoflurane under a gas anesthesia machine (RWD Life Science Co., China). The skin of the back scapula area was cut open and the mice were injected with PBS or 5ng/g/d CXCL13 into the BAT. The skin was carefully closed with surgical sutures.

All procedures involving animals were conducted according to the guidelines set forth by the Institutional Animal Care and Use Committee (IACUC) of the Second Xiangya Hospital of Central South University, Changsha, Hunan, China.

### 2.3 Indirect calorimetry

Energy expenditure of the ND and HFD mice (CXCL13 group vs. PBS group) was determined by Oxymax/CLAMS metabolic cages (Columbus Instruments, USA) at standard room temperature conditions. The oxygen consumption (VO2), carbon dioxide production (VCO2), respiratory exchange ratio (RER, ratio of VCO2 to VO2), energy expenditure (heat), and food intake (feed) were monitored. The CLAMS metabolic cages collect data about every 15 minutes. Every 3-4 individual points were then averaged to provide a 1-hour reading measurement. Data is reported as mean ± SEM of light and dark cycles.

### 2.4 Isolation of stromal vascular fraction

Adipose tissues were cut into pieces and digested with type II collagenase (Sigma, USA) on a constant temperature air shaker at 37°C. After digestion, the lysate was washed, and centrifuged. The upper layer containing mature adipocytes and the bottom sediment containing SVF were collected for further analyses.

### 2.5 Primary brown adipocyte culture and treatment

The freshly collected BAT between the scapulae region of the mice was washed with pre-chilled high-sugar DMEM (Gibco, USA). Tissues were cut, digested, then filtered, centrifuged, and suspended with DMEM containing 20% fetal bovine serum (FBS, Gibco, USA) and 1% penicillin/streptomycin (P/S, sigma, USA). Cells were plated, cultured, and induced to differentiate according to our previously reported procedures (17). The initial differentiation medium was supplemented with 0.5 mM isobutylmethylxanthine, 0.5 μM dexamethasone, 0.125 mM indomethacin, 1 nM T3 (Sigma, USA), and 20 nM insulin (Novo Nordisk, China) for 48 hours and then replaced by differentiation medium containing the same concentration of T3 and insulin for further culture until 5-6 days. For treatment, Forskolin (HY-15371, MedChemExpress Inc., China) was added to preadipocytes and differentiated adipocytes were cocultured with M2 macrophages.

### 2.6 Culture and treatment of bone marrow-derived macrophages

BMDM were collected and cultured using the methods previously reported (18). Cells well brushed from bones, filtered, plated, and cultured for 8-10 days with 20 ng/mL M-CSF and 10% FBS in the DMEM. Macrophages were treated with 100 ng/mL LPS or 100 ng/mL IL4 for 12h. For the Transwell migration assay, DMEM or pre-adipocyte cell medium (CM) was added to lower chambers with CXCL13, anti-CXCL13 antibody or anti-goat IgG (ab6885, Abcam, USA), and the upper chambers contained serum-free DMEM with approximately 2*10^5 BMDM or RAW264.7 suspension. The chambers were incubated at 37 °C overnight. The cells were fixed with formaldehyde (4%), stained with hematoxylin, and observed under a light microscope (Olympus Corp. Japan). For co-culture assay, the lower chambers were plated with mature adipocytes, and the upper chambers were plated with BMDM suspension. The chambers were incubated at 37 °C for 2 days with DMEM containing 20% FBS.

### 2.7 RAW 264.7 cell culture

RAW 264.7 macrophages were grown in DMEM supplemented with 10% FBS and 1% P/S. Cells were plated and starved when reached 80% confluence for 12 hours. Cells were treated with 100 ng/mL LPS or 100 ng/mL IL4 for 12h. The Transwell migration assay was performed as described above.

### 2.8 Flow cytometry

Single cells of SVF fraction were resuspended with PBS and stained with zombie NIR™ dye (#423105, Biolegend, USA), blocked with FcX Blocking CD16/32 (#156604), and dyed with a mix of surface marker antibodies. Cells were washed with cell staining buffer (#554657, BD Biosciences, USA), suspended, and transferred to a flow tube. Data was collected on a NovoExpress flow cytometer (Agilent, USA) and analyzed using Flowjo_V10 software (Tree Star).

### 2.9 Real-time RT-PCR

Total RNAs were extracted with Magzol reagent (R4801-01, Magen, China) following the manufacturer’s protocol. Reverse transcription of mRNA was performed using an Evo M-MLV Reverse Transcription Premix Kit (AG11728, Accurate Biology, China). Real-time PCR was performed using Hieff^®^ qPCR SYBR Green Master Mix (11202ES03, Yeasen, China) on a 7900HT Fast Real-Time PCR System (Applied Biosystems, USA). Primers are listed in **Supplementary Table S1**.

### 2.10 Western blot

Adipose tissue lysates were mixed in the 5×SDS loading buffer and heated to 100°C for 10 min. Samples were run on a 12% Bis-Tris criterion gel (Bio-Rad, Hercules, CA, USA), incubated with primary antibodies overnight, and then incubated with the second anti-rabbit IgG (W4018, PROMEGA, USA) or anti-goat IgG. Signals were detected using the ChemiDOCTMXRS+ and the Image LabTM system (BIO-RAD, USA).

### 2.11 ELISA

The concentrations of CXCL13 (R&D Systems, USA) and TNFα (4A Biotech., Ltd, China) were measured using ELISA kits according to the manufacturer’s instructions. All samples and standards were put into the corresponding wells. A biotinylated antibody working solution was added and incubated with an enzyme conjugate working solution for 30 min. The color developer was added in the dark, and the OD450 value was measured using a multifunctional enzyme analyzer (Perkin Elmer, USA).

### 2.12 Plasma lipid measurements

The plasma levels of triglyceride (TG) and cholesterol (T-CHO) were measured by Triglyceride assay kit (A110-1-1, Nanjing Jiancheng Bio Inc., China) and Total cholesterol assay kit (A111-1-1), respectively, according to the manufacturer’s instructions.

### 2.13 Immunohistochemical staining

Tissues were fixed in 4% paraformaldehyde and embedded in paraffin. For immunohistochemistry (IHC) staining, tissue section slides were incubated with primary antibodies of CD206 (ab64693, Abcam, USA), UCP1 (GB112174, Servicebio Inc., China), or TNFα (AF06294, AiFang Biological Inc., China) overnight at 4°C, then with the secondary antibodies at room temperature for 30 min. DAB was used for color development. The IHC staining was scanned and quantified with ImageJ 2.0 (NIH, USA).

### 2.14 Datasets reanalysis

The RNA-seq raw data were downloaded from https://www.ncbi.nlm.nih.gov/, including GEO Datasets: GSE77534, GSE135391, GSE86338, GSE207705, and GSE181123. All datasets were analyzed with Rstudio software (Posit Software, USA). The “DESeq2” or “limma” packages were used for differential gene analysis, and the upregulated genes were defined as p-value <0.05 and log2fold change > 2 between cold and control groups in BAT. The “ggplot2” and “ggVennDiagram” installation packages were used for Venn chart mapping.

The single-cell RNA-sequencing data was downloaded from GEO datasets GSE207706. Data was input and integrated into the R program “Seurat” and was filtered by percent.mt <0.2 & nFeature RNA >200.

### 2.15 Statistical analysis

All data were shown as mean ± SEM. Comparison between two groups was conducted using Student’s two-tailed t-test and multiple groups were analyzed using one-way analysis of variance (ANOVA) (v19.0, SPSS Inc., USA). The p-value less than 0.05 was considered to be statistically significant.

## 3 Results

### 3.1 Identification of CXCL13 as an elevated chemokine in brown adipose tissue in response to cold stimulation

**Figure 1:**
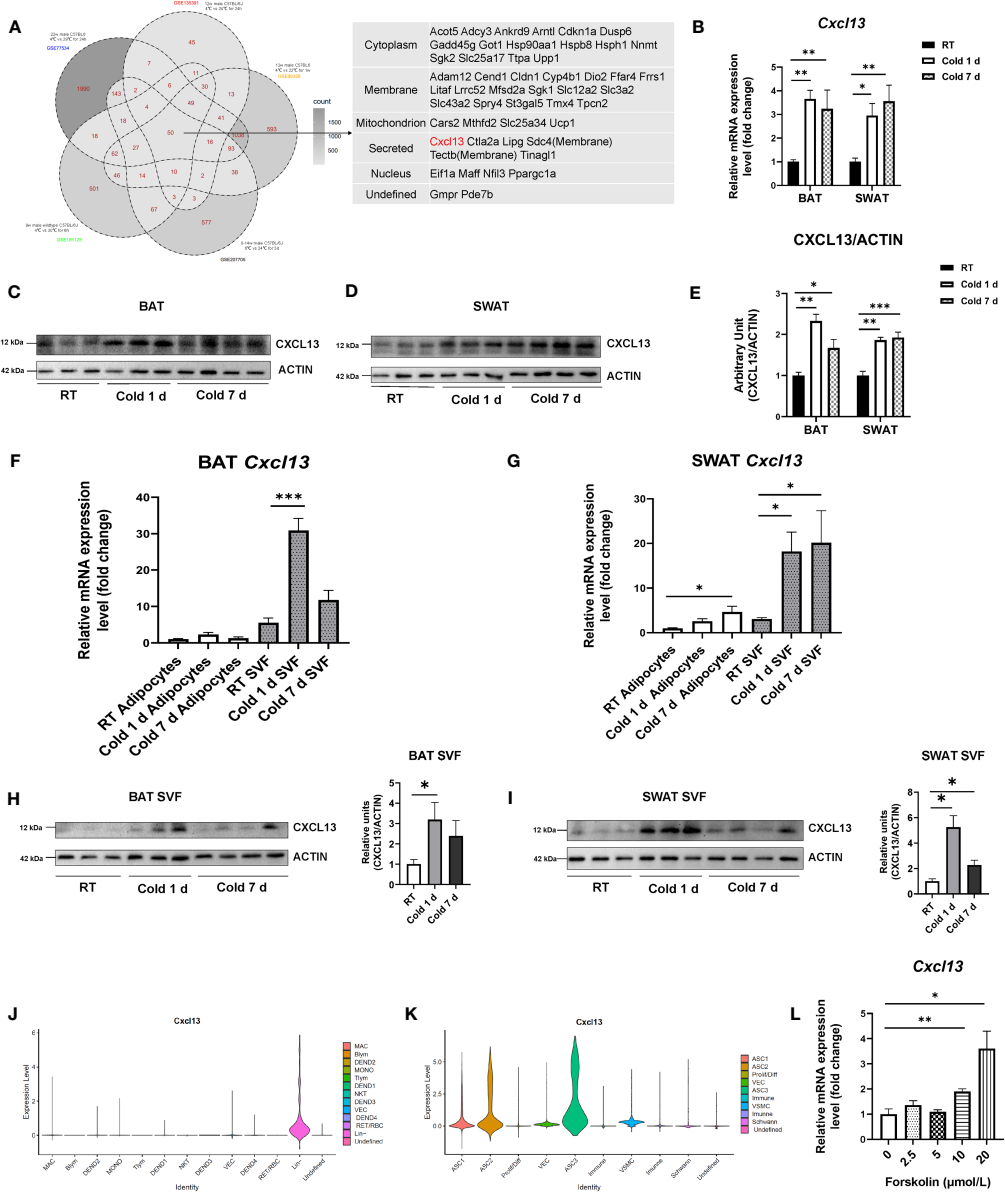
Identification of CXCL13 as an elevated chemokine in brown adipose tissue in response to cold stimulation. **(A)** Venn chart of RNA-seq database to screen candidate upregulated molecules preferentially expressed in the BAT in response to thermogenic activation (right) and the list of genes upregulated in all five databases (left). **(B)** Eight-week-old C57BL6/J male mice were exposed to the cold (6°C) for 1 day or 7 days while the control mice were set at the 22 °C room temperature (RT). mRNA levels of the *Cxcl13* gene in the BAT and SWAT (n=4-6/group) were analyzed. Western blot analysis of CXCL13 protein in the BAT **(C)** and the SWAT **(D)** of the mice exposed to cold or RT (n=3-4/group). **(E)** Quantification of CXCL13 protein in **(C, D)**. Expression of Cxcl13 mRNA in SVF and mature adipocytes of the BAT **(F)** and SWAT **(G)** (n=4-6/group). Western blot analysis of CXCL13 protein in the SVF of BAT **(H)** and the SWAT **(I)** of the mice exposed to cold or RT (n=3-4/group). **(J)** Violin plots displaying the log2 expression levels for *Cxcl13* in Lin+ cells. MAC, macrophage; MONO, monocyte; DEND, dendritic cell; NKT, natural killer T-cell; Tlym, T lymphocyte; Blym, B lymphocyte; RET, reticulocyte; NEUT, neutrophil; VEC, vascular endothelial cell; Lin-, lineage-negative cells; RBC, red blood cell. **(K)** Violin plots displaying the log2 expression levels for *Cxcl13* in Lin- cells. ASC, adipose tissue stromal cell; VEC, vascular endothelial cell; VSMC, vascular smooth muscle cells; Prolif/Diff, proliferating/differentiating cells. **(L)** The mRNA expression of *Cxcl13* in primary brown adipocytes was treated with different concentrations of Forskolin for 6 hours (n=3-5/group). Data were presented as mean ± S.E.M. *p< 0.05, **p<0.01, ***p<0.001.

Since rodents may respond differently to diverse condition of cold stimulation, such as duration of cold exposure (acute vs. chronic) and different temperatures (room, thermoneutral vs. cold), to identify factors that may be involved in thermogenesis during adaptive thermogenesis in the BAT, we have selected and analyzed 5 different datasets (GEO: GSE135391, GSE86338, GSE207705, GSE181123, GSE77534) generated under different cold conditions (19–23). All the data was collected from the BAT of male C57BL/6J mice under either room (24°C) or thermoneutral (29-30°C) temperature versus cold (4-6°C) temperature for different periods (ranging from 6 hrs to 1 wks) (**Figure 1A**, left). Fifty significantly up-regulated candidates in all the datasets were detected. Genes that are expressed in the cytoplasm, mitochondria, or cell membrane and nucleus are listed in **Figure 1A** (right panel). Among the candidate genes, the *Cxcl13* was the only chemokine that we assumed might act as a brown adipokine and function during cold stimulation.

To confirm the up-regulation of CXCL13 in the adipose tissues during cold exposure, the 8-week-old C57BL/6J mice were put at 6°C for 1 day or 7 days. The results showed that cold stimulation for either 1 or 7 days significantly increased the mRNA levels of *Cxcl13* in both BAT and SWAT (**Figure 1B**). In other tissues of C57BL/6J mice under cold stimulation, *Cxcl13* had no significant change in the heart, kidney, liver, brain, and muscle, but increased in the intestine and spleen (**Figure S1A**). The expression of CXCL13 receptor *Cxcr5* was also significantly induced in the BAT during both 1-day and 7-day cold exposure (**Figure S1B**). Cold-stimulation-increased CXCL13 expression was also confirmed at protein levels in SWAT and BAT (**Figures 1C-E**), but not in the serum (**Figure S1C**), indicating that increased CXCL13 might act inside the BAT tissue. We also measured CXCL13 expression in VAT but observed no significant changes (**Figures S1D, E**).

To investigate in which the components of adipose tissue CXCL13 expression was elevated, we isolated the SVF and mature adipocytes of adipose tissues from mice under cold exposure. Our results showed that during both 1- and 7-day cold exposure, the level of *Cxcl13* in the SVF component was significantly higher than that in the mature adipocytes of BAT (**Figure 1F**). A similar pattern was observed in the SVF and mature adipocytes of the SWAT (**Figure 1G**). Furthermore, the elevated CXCL13 protein levels after 1 or 7 days of cold stimulation were also shown in the SVF component of BAT and SWAT (**Figures 1H, I**), which is consistent with the mRNA level.

To identify the cell lineage in the SVF of BAT that expresses CXCL13, we have analyzed single-cell RNA-sequencing (scRNA-seq) data from GEO Datasets (GSE207706) and identified 13 clusters according to the marker genes reported by Rayanne B Burl et al. (**Figures S1I, J**) (22). The *Cxcl13* was mainly expressed in Lin negative (Lin-) cells, including adipose stromal cells (ASCs), vascular cells, and proliferating/newly differentiating adipocytes (**Figure 1J**). We further analyzed the Lin- cells in the datasets and identified 10 clusters (**Figures S1K, L**). The result showed that *Cxcl13* is mainly expressed in the innate PDGFRA+ ASCs including ASC1, ASC2, and ASC3 (**Figure 1K**).

To mimic cold stimulation on ASCs *in vitro*, we treated the SVF-derived brown pre-adipocytes with different concentrations of Forskolin, a potent adenylate cyclase activator that induces the cAMP/PKA signaling pathway (24). The results showed that Forskolin significantly stimulated the expression of *Cxcl13* and *Cxcr5* in brown pre-adipocytes (**Figure 1L** and **Figure S1M**). Taken together, our data suggests that CXCL13 expression was elevated in BAT and SWAT under cold stimulation and the elevation of CXCL13 occurred primarily in the SVF of adipose tissues.

### 3.2 CXCL13 induced BAT activation and promoted thermogenesis in lean mice

To investigate the effect of CXCL13 in adipose tissue, we conducted local injections of CXCL13 in the BAT of C57BL/6J mice fed with a normal diet. We tested different concentrations of CXCL13, namely 1, 2.5, and 5 ng/g body weight (BW) (**Figures S2A, B**). The protein levels of CXCL13 and UCP1 were upregulated significantly, based on these results, we selected the concentration of 5 ng/g BW CXCL13 for further experiments. Additionally, to ensure the injected CXCL13 function in the BAT but not in circulation or other tissues, we measured the levels of CXCL13 in serum, BAT, SWAT, and VAT after injection (**Figures S2C, D**), and confirmed that the CXCL13 was significantly higher in the BAT but not in the SWAT, VAT, and serum.

**Figure 2:**
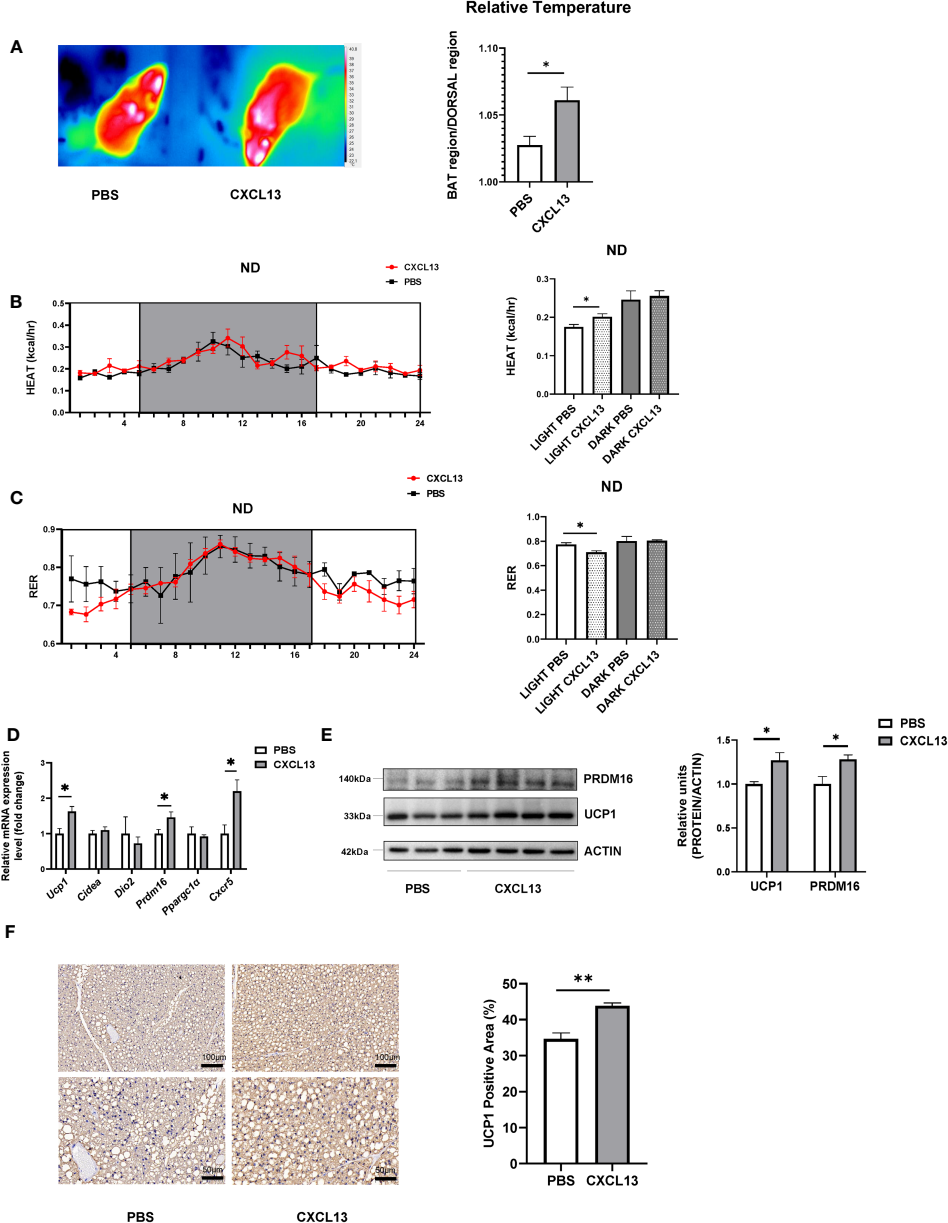
CXCL13 induced BAT activation and promoted thermogenesis in lean mice. Normal-diet-fed C57BL6/J male mice were treated with CXCL13 or PBS through local BAT injection for 3 days. **(A)** Representative thermal images (left) and surface temperature ratio of the interscapular region (right) in PBS and CXCL13 injected mice (n=4-5/group). **(B)** Heat production of PBS and CXCL13 injected mice. Values represent measurements of 24 hours (n=3-4/group, left). Quantitative measurements of heat production in the mice (right). **(C)** Respiratory exchange ratio (RER) of PBS and CXCL13 injected mice. Values represent measurements of 24 hours (n=3-4/group, left). Quantitative measurements of RER in the mice (right). **(D)** mRNA levels of thermogenic genes and Cxcr5 in the BAT (n=4-5/group). **(E)** Western blot analysis of UCP1 and PRDM16 protein in the BAT (n=3-4/group, left). Quantitative relative measurements of proteins to ACTIN (right). **(F)** Representative images of UCP1 immunohistochemical staining of the BAT from PBS and CXCL13 injected groups (left) and the statistical analysis (right, n=3/group). Data were presented as mean ± S.E.M. *p< 0.05, **p<0.01.

After 3-consecutive-days of CXCL13 or PBS vehicle injection, the relative surface temperature of the scapular region was measured using thermography. The results have shown that the CXCL13 injection significantly increased the relative temperature of the mice when compared to vehicle control mice (**Figure 2A**), although the BW and rectal temperature were comparable between the two groups (**Figures S2E, F**). We then evaluated heat production and respiratory exchange ratio (RER) using indirect calorimetry measurements in metabolic cages (25). CXCL13 injected mice showed higher heat production (**Figure 2B**), VO2 consumption (**Figure S2G**), and lower RER (**Figure 2C**) during the daytime, indicating increased energy expenditure, while there was no significant change in the food intake (**Figure S2I**). In line with the higher surface temperature, the CXCL13 injection increased mRNA expression of thermogenic genes (*Ucp1* and *Prdm16*) and *Cxcr5* expression in the BAT (**Figure 2D**), but not in SWAT (**Figure S2J**). The western blotting and immunohistochemical staining also showed significantly upregulated UCP1 protein levels (**Figures 2E, F**), indicating that CXCL13 can promote BAT thermogenesis in lean mice.

### 3.3 CXCL13 promoted thermogenesis and enhanced cold tolerance in diet-induced obese mice

**Figure 3:**
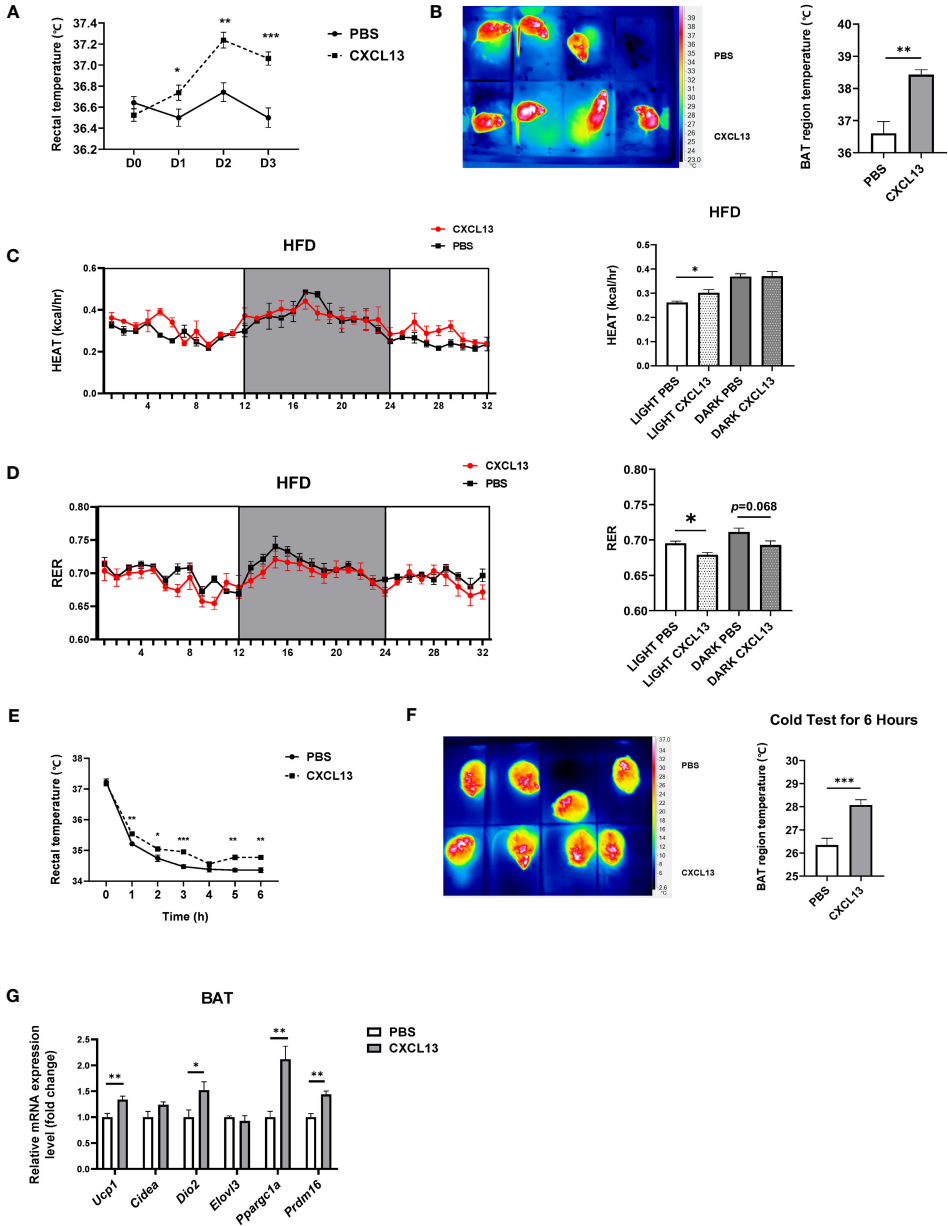
CXCL13 promoted thermogenesis and enhanced cold tolerance in diet-induced obese (DIO) mice. High-fat diet (HFD)-induced obese C57BL6/J male mice were locally injected with CXCL13 or PBS in the BAT for 3 days. **(A)** Changes of rectal temperature during 3 days of PBS or CXCL13 injection (n=6-8/group). **(B)** Representative thermal images (left) and BAT region temperature in PBS and CXCL13 injected mice (right) (n=6-8/group). **(C)** Heat production of PBS and CXCL13 injected obese mice (n=3-4/group, left) and quantification of the light and night period (right). **(D)** RER of PBS and CXCL13 injected obese mice (n=3-4/group, left) and quantification of the light and night period (right). **(E)** Changes in rectal temperature during a 6-hour cold tolerance test (n=6-8/group). **(F)** Representative thermal images (left) and BAT region temperature in PBS and CXCL13-injected mice (right) after 6 hours of cold exposure (n=4/group). **(G)** mRNA levels of thermogenic genes in the BAT (n=6-8/group). *p < 0.05, **p <0.01, ***p <0.001.

To further explore the effects of CXCL13 in obese mice, the DIO mice were BAT injected with CXCL13 for 3 days. The rectal temperature of the CXCL13 group was significantly higher compared with that of the control group (**Figure 3A**). Similar to observations in normal-diet-fed mice, the surface temperature of the scapular region significantly increased in CXCL13-injected obese mice (**Figure 3B**). CXCL13-injected obese mice also showed higher heat production (**Figure 3C**) and lower RER (**Figure 3D**) during the daytime, suggesting elevated energy expenditure and lipids utilization.

To activate the BAT function, we placed the mice at a cold temperature (6°C) for six hours and measured both rectal and surface body temperature. We have found that mice with CXCL13 injections could maintain both their rectal and surface body temperatures at higher levels (**Figures 3E, F**), suggesting enhanced cold tolerance.

Previous studies reported that during adaptive thermogenesis under cold conditions, activated BAT can burn lipids to produce heat, thus decreasing plasma TG and cholesterol (T-CHO) levels (26, 27). We have determined whether CXCL13 injection could alter TG and T-CHO levels in obese mice, and the results have shown that CXCL13 could decrease T-CHO levels after cold tests (**Figure S3F**). Consistently, expression of thermogenic genes (*Ucp1*, *Dio2*, *Ppargc1α*, and *Prdm16*) was elevated after a 3-day CXCL13 injection in the obese mice after 6-hour cold stimulation (**Figure 3G**). Overall, CXCL13 could promote thermogenesis, enhance cold tolerance, and decrease lipid levels in obese mice during cold stimulation.

### 3.4 CXCL13 induced M2 macrophage migration in brown adipose tissue

**Figure 4:**
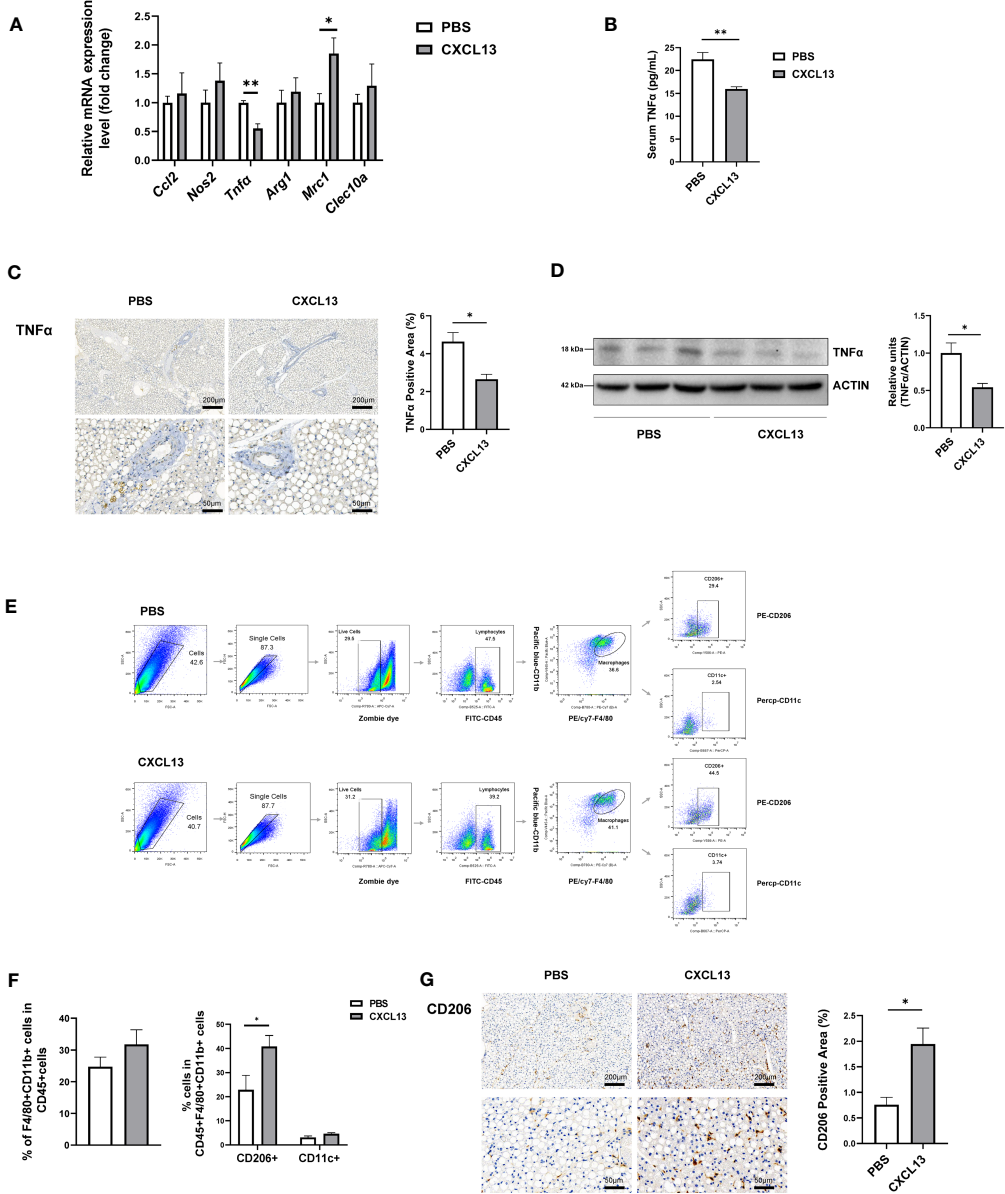
CXCL13 induced M2 macrophage migration in brown adipose tissue. Normal-diet-fed C57BL6/J male mice were treated with CXCL13 or PBS for 3 days using BAT injection. **(A)** Relative mRNA levels of genes indicative of M2 and M1 macrophage genes in the BAT (n=3-5/group). **(B)** Serum TNFα level after BAT CXCL13 injection (n=5/group). **(C)** Representative images of TNFα immunohistochemical staining of the BAT (left) and the statistical analysis (right, n=3/group). **(D)** Western blot analysis of TNFα protein in the BAT (n=3/group, left). Quantitative measurements of TNFα proteins to ACTIN (right). **(E)** Flow cytometry gating schemes for subset distribution of macrophages in SVF of the BAT. **(F)** The percentage of F4/80+CD11b+ (total macrophage) within the CD45+ cells (left), and CD11c+ cells (M1 macrophage) or CD206+ cells (M2 macrophage) within the CD45+F4/80+CD11b+ population from the BAT (right) (n=4/group) **(G)** Representative images of CD206 immunohistochemical staining of the BAT and statistical analysis (n=3/group). *p < 0.05, **p <0.01.

The chemokine CXCL13 has been reported as a major regulator of the immune response and plays a key role in the pathophysiology of inflammatory, infectious, and lymphoproliferative diseases (8), so we have assumed that CXCL13 might promote thermogenesis through its effect on immune cells. To detect whether CXCL13 could influence immune cells in the BAT, we first examined immune cell-related genes (*Cd19*, *Cd5*, *F4/80*, *Cd3*) in the BAT after injection, but no significant changes were found in mRNA levels of immune cell markers (**Figure S4A**). Studies have shown that immune cells play an important role in brown fat activation and white fat browning via the recruitment of alternatively activated macrophages (28). Therefore, we have tested M1 (*Nos2*, *Tnfα*, *Ccl2*) and M2 (*Arg1*, *Clec10a*, *Mrc1*) macrophage genes, and found that the expression of pro-inflammatory gene *Tnfα* was decreased, whereas M2 macrophage marker gene *Mrc1* (also called *Cd206*) was elevated after 3 days of CXCL13 BAT injection (**Figure 4A**). The decreased TNFα protein levels were confirmed in both serum (**Figure 4B**) and the BAT (**Figures 4C, D**).

We then tested the proportion of macrophage in the SVF of BAT through flow cytometry. We gated CD45+ cells from live single cells as myeloid cells, and F4/80+ plus CD11b+ cells as macrophages, then CD206+ cells as M2 macrophages and CD11c+ cells as M1 macrophages (**Figure 4E**) (29). The results showed that there was a trend of increased total macrophages and a significantly increased proportion of M2 macrophages, but not M1 macrophages in the CXCL13-injected mice (**Figure 4F**). The proportions of T cells and B cells in the SVF of BAT showed no significant changes in the CXCL13 injected mice (**Figure S4B**). The immunohistochemical staining further verified the increase of CD206+ cells in the BAT (**Figure 4G**). These results have indicated that CXCL13 could promote M2 macrophage migration in the BAT.

### 3.5 CXCL13 down-regulated the expression of M1 macrophage pro-inflammatory gene and up-regulated the expression of M2 macrophage marker genes *in vitro*

**Figure 5:**
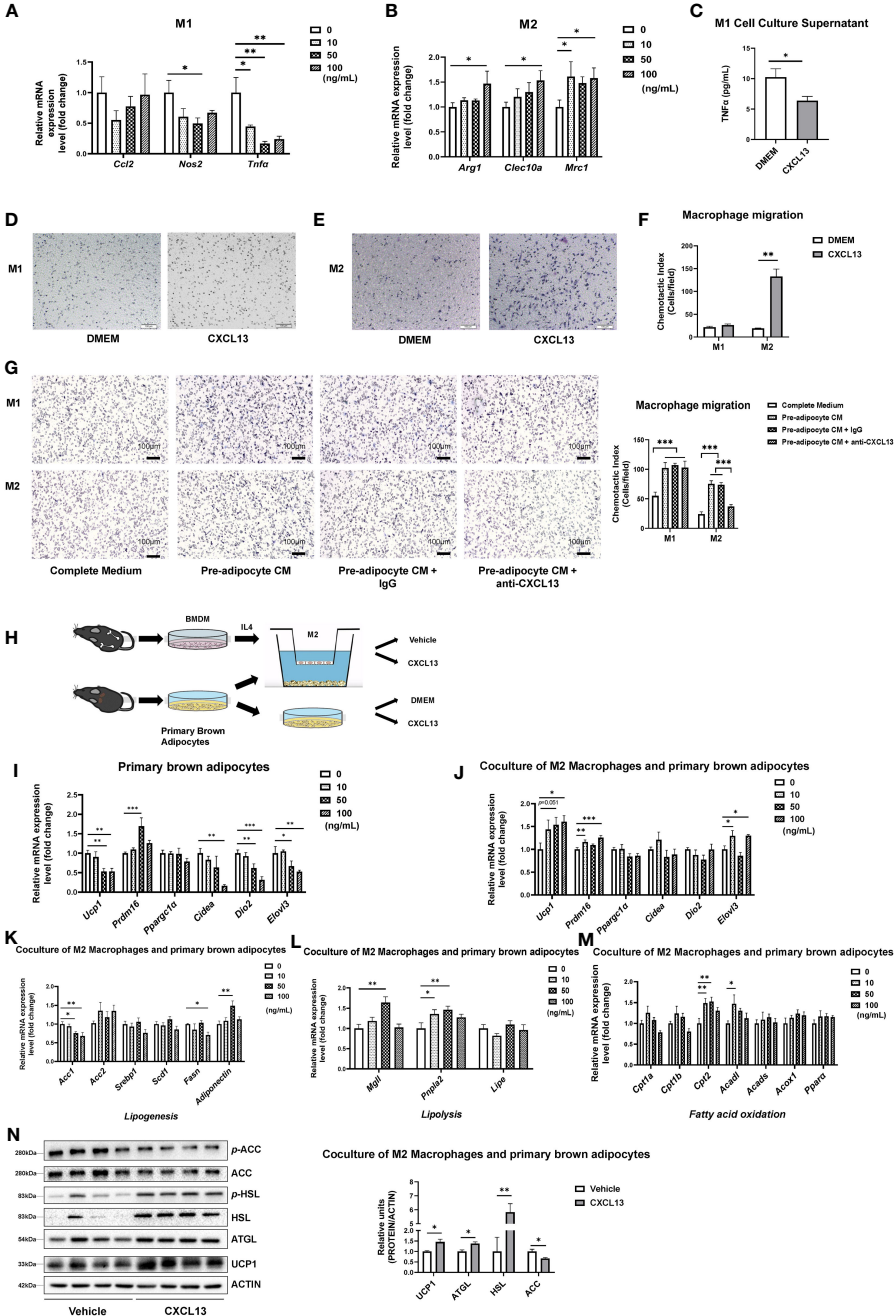
CXCL13 down-regulated the expression of the M1 macrophage pro-inflammatory gene and up-regulated the expression of M2 macrophage marker genes *in vitro*. Bone Marrow-derived Macrophages (BMDM) were cultured and treated with different dosages of CXCL13 for 12 h. **(A)** Activated M1 macrophages were treated with different concentrations of CXCL13 (n=3-4/group). **(B)** Activated M2 macrophages were treated with different concentrations of CXCL13 (n=4-6/group). **(C)** The TNFα content in the supernatant of M1 macrophages culture medium treated with 50 ng/mL CXCL13 (n=3-4/group). **(D)** M1 **(D)** and M2 **(E)** macrophages migration assay induced by 50 ng/mL CXCL13 or control medium. Cells were fixed and stained by hematoxylin. **(F)** Quantification analysis of cells in figures D and E (n=3/group). **(G)** M1 and M2 macrophages migration. M1 or M2 macrophages were cultured with complete medium (CM), pre-adipocyte CM, pre-adipocyte CM + IgG or pre-adipocyte CM + anti-CXCL13 for 20 hrs. Cells were fixed and stained by hematoxylin (n=3/group). **(H)** Diagram of the co-culture system of primary brown adipocytes and M2 macrophages. **(I)** mRNA levels of thermogenic genes in the brown adipocytes treated with different concentrations of CXCL13 (n=4-6/group). **(J)** mRNA levels of thermogenic genes in the primary brown adipocytes co-cultured with M2 macrophages and treated with different concentrations of CXCL13 (n=4-6/group). **(K)** mRNA levels of lipogenesis genes in the primary brown adipocytes co-cultured with M2 macrophages and treated with different concentrations of CXCL13 (n=4-6/group). **(L)** mRNA levels of lipolysis genes in the primary brown adipocytes co-cultured with M2 macrophages and treated with different concentrations of CXCL13 (n=4-6/group). **(M)** mRNA levels of fatty acid oxidation genes in the primary brown adipocytes co-cultured with M2 macrophages and treated with different concentrations of CXCL13 (n=4-6/group). **(N)** Western blot analysis of Lipogenic (ACC), lipolysis (HSL and ATGL), and thermogenic (UCP1) protein levels in the primary brown adipocytes co-cultured with M2 macrophages and treated with 50 ng/mL CXCL13 (n=4/group, left) Quantitative measurements of proteins to ACTIN (right). *p<0.05, **p<0.01,***p<0.001.

To further explore the function of CXCL13 in macrophages *in vitro*, we first induced primary bone marrow cells into M1 or M2 macrophages using LPS or IL4 treatment (**Figure S5A**), respectively and then treated them with CXCL13 recombinant protein. Our results have shown that CXCL13 treatment caused decreases in the expression of *Tnfα* and led to an increase in the expression of M2 macrophage marker genes (*Arg1*, *Cleac10a*, *Mrc1*) (**Figures 5A, B**). There was also a decrease in TNFα secretion in the culture supernatant of the M1 macrophage under the treatment of CXCL13 (**Figure 5C**).

Next, we tested the ability of migration of BMDM treated by recombinant CXCL13 and found that CXCL13 could significantly induce the migration of chemotactic M2 macrophages, but not M1 macrophages (**Figures 5D-F**). To investigate whether pre-adipocyte derived CXCL13 could recruit macrophages, we conducted an experiment using the cell medium (CM) of pre-adipocytes with either anti-goat IgG or anti-CXCL13 antibody to chemotaxis M1 or M2 macrophages in BMDM (**Figure 5G**) and RAW264.7 cell line (**Figure S5B**). Our results showed that the CM of pre-adipocytes could significantly induce both M1 and M2 macrophages, while the anti-CXCL13 antibody inhibited the migration of M2 macrophages, further confirming the effect of CXCL13 on M2 macrophage migration.

To test whether the activated M2 macrophage mediates the action of CXCL13 on BAT thermogenesis, we have treated primary brown adipocytes with recombinant CXCL13 or the vehicle control in the presence or absence of M2 macrophages (**Figure 5H**). Our results have shown that treatment of CXCL13 alone decreased thermogenetic gene expressions in brown adipocytes whereas the presence of M2 macrophage could promote the up-regulation of thermogenic genes (*Ucp1*, *Prdm16*, and *Elovl3*) when treated by CXCL13, suggesting that M2 macrophage might mediate the action of CXCL13 on thermogenic gene expression (**Figures 5I, J**). Besides, when co-culture M2 macrophage and brown adipocytes, CXCL13 treatment could decrease the mRNA levels of lipogenesis genes (*Acc1* and *Fasn*, **Figure 5K**), but increase the expression of lipolysis genes (*Mgll* and *Pnpla2*, **Figure 5L**) and fatty acid oxidation genes (*Cpt2* and *Acadl*, **Figure 5M**), supporting the elevated thermogenic function (30). The protein levels of lipogenesis (ACC), lipolysis (HSL and ATGL), and thermogenesis (UCP1) were consistent with the mRNA levels after coculturing of M2 macrophages and brown adipocytes (**Figure 5N**). However, there were no significant changes when brown adipocytes were treated with CXCL13 alone (**Figure S5C**). Taken together, our data have suggested that CXCL13 could promote BAT thermogenesis through recruitment and activation of M2 macrophages and, in the meantime, inhibit inflammation by decreasing TNFα levels.

## 4 Discussion

In this study, we have discovered that CXCL13 derived from the SVF fraction of the BAT inhibited inflammation and promoted thermogenesis through recruiting and activating M2 macrophages. The BAT CXCL13 administration increased heat production and enhanced cold tolerance in obese mice. We illustrated the immune function of CXCL13 in adipose tissue and provided a promising therapeutic target from ASCs on obesity.

In mammals, BAT is a major site for regulating non-shivering thermogenesis to maintain body temperature. BAT also secretes various brown adipokines to regulate energy homeostasis (31). The SVF components of the BAT contain ASCs, precursor adipocytes, endothelial cells, hematopoietic cells, and neural cells (32). We found that elevated chemokine CXCL13 after cold stimulation occurred mainly in the SVF of both BAT and SWAT. The results were further validated by scRNA-seq, which confirmed that Lin- ASCs might be the source of CXCL13 in the BAT. The scRNA-seq datasets we reanalyzed were from the BAT of C57 mice in RT and cold exposure conditions based on the clusters defined by Rayanne B. Burl et al. (22). In their study, cold exposure could significantly increase the ASC1 cluster, while we found that *Cxcl13* mainly expressed in the ASC2 and ASC3 clusters, the resident ASCs during cold exposure. In another study, Nahmgoong et al. identified the Bst2^high^ (bone marrow stromal cell antigen 2) ASC cluster in sWAT, which expressed high levels of beige related markers *Tmem26*, *Il33*, *Ucp1*, and also *Cxcl13* (33). The Bst2^high^ ASC cluster is mainly located near lymph nodes and up-regulated after cold exposure (33). These studies and our results suggest that ASC derived CXCL13 could respond to cold stimulation.

Besides, ASCs in the WAT could secrete prostaglandin E2 (PGE2), leukemia inhibitory factor (LIF), and kynurenine to suppress inflammation, as well as vascular endothelial growth factor (VEGF) and insulin-like growth factor-1 (IGF-1) to promote tissue repair and regeneration, thus attributing to their therapeutic potential for the treatment of diseases (34). Besides cytokines, exosomes from ASCs of VAT could prevent obesity and attenuate inflammation by promoting M2 macrophages polarization (35). Most studies are associated with the function of ASCs in the WAT, while studies on the secreted functions of ASCs in the BAT are limited. Our study suggested that chemokine CXCL13 might be secreted from ASCs of the BAT under cold stimulation and promoted brown adipocyte activity in a paracrine manner.

We found that the elevation of CXCL13 in the BAT could increase regional scapular temperature and heat production, but decrease RER levels, indicating the improvement in energy expenditure and lipid utilization in both lean and obese mice. In response to cold exposure, CXCL13-injected obese mice also maintained a relatively higher body temperature and displayed an increased level of thermogenic gene expression in the BAT suggesting elevated thermogenesis and cold tolerance. Some brown adipokines have been reported to promote thermogenesis or energy expenditure in a UCP1-dependent manner. For instance, BAT-enriched adipose-secreted signaling protein (Adissp) can increase energy expenditure, UCP1 expression, and insulin sensitivity via cAMP/PKA signaling pathways (36). A chemokine CXCL14 can also recruit alternatively activated (M2) macrophages and promote adipose tissue browning (37). In our study, we have explored the function of CXCL13 in thermogenesis using short-term BAT injection and identified CXCL13 as a novel brown adipokine that could promote thermogenesis and energy expenditure in mice.

Obesity is associated with the recruitment of monocytes into adipose tissues, which leads to differentiation into pro-inflammatory M1 macrophages. These M1 macrophages produce inflammatory cytokines such as TNFα to block insulin action in adipocytes, impair preadipocyte differentiation, and induce apoptosis of brown adipocytes. In contrast, anti-inflammatory M2 macrophages produce anti-inflammatory cytokines, such as interleukin (IL)-4 and IL-10, to promote insulin sensitivity and adipocyte differentiation in lean individuals (38, 39). Elevated levels of TNFα or IL-1β are also associated with reduced energy expenditure (40), whereas suppressing TNFα significantly reduces brown adipocyte apoptosis and increases beta3-adrenoreceptor and UCP1 expression in the BAT of obese mice with obesity (38). It has been found that TNF-α and IL-10 could control the expression of the *Cxcl13* gene in human blood monocyte-derived macrophages and alveolar-derived macrophages through activation of NF-κB and JAK/STAT signaling pathways, respectively (12). Our results demonstrated that CXCL13 might have a negative feedback inhibitory effect on the expression of TNFα in M1 macrophages in the BAT, which deserves further investigation in the future.

Cold stimulation can significantly increase CD206+ macrophage proportion and decrease the CD11c+/CD206+ ratio in the BAT and sWAT (41–43). Cold stress stimulates elevation of some cytokines, such as IL-4 and IL-13 secreted by eosinophils, in adipose tissue to induce selective activation of M2 macrophages, which in turn promotes lipolysis, beiging of white adipocytes and thermogenesis of brown fat, thus improveing the metabolic homeostasis (44). Except for anti-inflammatory cytokine secretion, M2 macrophages also promote thermogenesis through the Slit3-sympathetic neuron axis (45). However, the mechanism of M2 macrophage-promoting thermogenesis remains unclear. Some regulatory molecules from adipose tissues that induce M2 macrophage activation under cold stimulation have been reported, such as CXCL14 (37), adipocyte ceramides (46), adiponectin (43), and FGF21 (42). Our study has found that ASCs-derived CXCL13 could induce M2 macrophage migration, which might partly be the underlying mechanism of cold-induced M2 macrophage migration in the BAT.

CXCL13 is well known for B1 lymphocyte chemotaxis in fat-associated lymphoid clusters (FALCs) through the CXCL13-CXCR5 axis (47–49). The main functional study of CXCL13 currently lies in its important role in lymph node formation and development (49), however, its role in the chemotaxis of immune cells of the BAT is unclear. In our studies, we discovered that, as a chemokine, CXCL13 induced M2 macrophages migration in the BAT. Recent studies in tumors, such as multiple myeloma and glioblastoma, have shown positive relations between CXCL13 and CD206+ M2 macrophages (10, 50), supporting the notion that CXCL13 has chemotaxis to CD206+ M2 macrophages in the BAT which may play a beneficial role in brown adipocytes thermogenesis.

**Figure 6:**
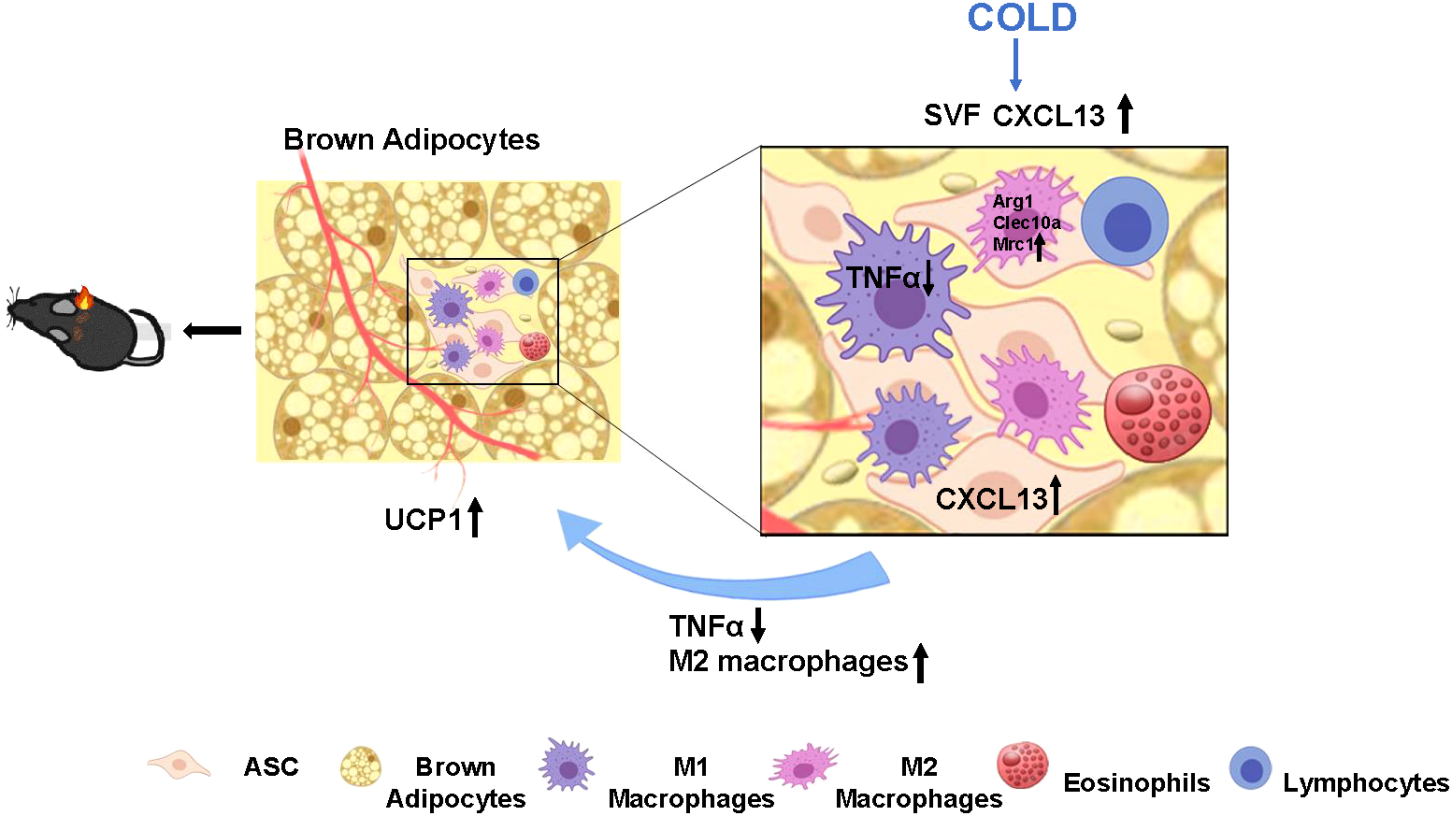
Proposed model of cold elevated CXCL13 expression in thermogenesis of brown adipose tissue in mice. Cold exposure induces elevation of CXCL13 in the adipose tissue stromal cell (ASC) of SVF. CXCL13 can promote brown adipocyte thermogenesis by inducing M2 macrophage activation and marker gene (*Arg1*, *Clec10a*, *Mrc1*) expression, and promoting cellular migration. CXCL13 can also suppress inflammation by down-regulating TNFα expression in M1 macrophages. The upward arrows represent up-regulation and the downward arrows represent down-regulation.

To summarize, we have reported that cold-stimulated CXCL13 secretion promotes thermogenesis in the BAT by inhibiting the inflammatory cytokine TNFα and promoting the recruitment of M2 macrophages to improve cold tolerance and energy expenditure in obese mice (**Figure 6**). CXCL13 administration could be a novel therapeutic strategy for obesity.

## Data availability statement

The data presented in the study are deposited in the NCBI GEO repository, accession numbers GSE77534, GSE135391, GSE86338, GSE207705, GSE181123 and GSE207706.

## Ethics statement

The animal study was approved by Institutional Animal Care and Use Committee (IACUC) of the Second Xiangya Hospital of Central South University, Changsha, Hunan, China. The study was conducted in accordance with the local legislation and institutional requirements.

## Author contributions

LX: Data curation, Formal Analysis, Writing – original draft. HW: Data curation, Formal Analysis, Writing – review & editing. DW: Data curation, Formal Analysis, Writing – review & editing. FZ: Data curation, Formal Analysis, Writing – review & editing. WC: Data curation, Formal Analysis, Writing – review & editing. YY: Data curation, Formal Analysis, Writing – review & editing. FH: Writing – review & editing, Supervision.
