# Supplementary material for: CXCL13 promotes thermogenesis in mice via recruitment of M2 macrophage and inhibition of inflammation in brown adipose tissue
Source: Front Immunol. 2023 Oct 23;14:1253766. doi: 10.3389/fimmu.2023.1253766 (PMC10627189; doi:10.3389/fimmu.2023.1253766)
Supplement: Supplementary file 1 [file DataSheet_1.docx]

Supplementary Material

CXCL13 Promotes Thermogenesis in Mice via Recruitment of M2 Macrophage and Inhibition of Inflammation in Brown Adipose Tissue

Lijun Xie, Huiying Wang, Dan Wu, Feng Zhang, Wei Chen, Yuqing Ye, Fang Hu*

*** Correspondence:** Fang Hu: [hu_fang98@csu.edu.cn](mailto:hu_fang98@csu.edu.cn)

# Supplementary Figures and Tables

# Supplementary Table

TableS1. Primers in this study.

| **Primer** | **Primer sequence** | |
| --- | --- | --- |
| *β-actin* | Forward | AGCCATGTACGTAGCCATCC |
| *β-actin* | Reverse | CTCTCAGCTGTGGTGGTGAA |
| *mRPS18* | Forward | CGCCATGTCTCTAGTGATCC |
| *mRPS18* | Reverse | GGTCGATGTCTGCTTTCCTC |
| *Cxcl13* | Forward | ATTCAAGTTACGCCCCCTG |
| *Cxcl13* | Reverse | TTGGCACGAGGATTCACAC |
| *Cxcr5* | Forward | TGGCCTTCTACAGTAACAGCA |
| *Cxcr5* | Reverse | GCATGAATACCGCCTTAAAGGAC |
| *Ucp1* | Forward | TACCAAGCTGTGCGATGTCCA |
| *Ucp1* | Reverse | GCACACAAACATGATGACGTTCC |
| *Ppargc1a* | Forward | CACTGACAGATGGAGCCGTGA |
| *Ppargc1a* | Reverse | TGTTGGCTGGTGCCAGTAAGAG |
| *Prdm16* | Forward | ATGCTGACGGATACAGAGGTG |
| *Prdm16* | Reverse | TCATCACAGGAACACGCTACA |
| *Cidea* | Forward | AAAGGGACAGAAATGGACACC |
| *Cidea* | Reverse | TACATCGTGGCTTTGACATTG |
| *Dio2* | Forward | AGTCTTTTTCTCCAACTGCCTCT |
| *Dio2* | Reverse | GGAGCATCTTCACCCAGTTTA |
| *Il10* | Forward | GCTCTTACTGACTGGCATGAG |
| *Il10* | Reverse | CGCAGCTCTAGGAGCATGTG |
| *Ccl2* | Forward | GCTCAGCC AGATGCAGTTA |
| *Ccl2* | Reverse | CTGCTGGTGATCCTCTTGTAG |
| *Nos2* | Forward | CCACGGACGAGACGGATAG |
| *Nos2* | Reverse | AAGGCAGCGGGCACAT |
| *Tnfα* | Forward | CCCCAGTCTGTATCCT |
| *Tnfα* | Reverse | TCTTATCCAGCCTCAT |
| *Arg1* | Forward | GCTTCGGAACTCAACG |
| *Arg1* | Reverse | CTGGGATACATACTTACTGG |
| *Clec10a* | Forward | CAGAATCGCTTAGCCAATG |
| *Clec10a* | Reverse | AGTCCGTGTCCGAACCAG |
| *Mrc1* | Forward | GCTTCCGTCACCCTGTATGC |
| *Mrc1* | Reverse | TCATCCGTGGTTCCATAGACC |
| *Cd19* | Forward | AATATGTCCAGCCGCTAC |
| *Cd19* | Reverse | GTGTTTCCCAGTTTGTGC |
| *Cd5* | Forward | ACAGAGCCTACGAGCAACC |
| *Cd5* | Reverse | GTGTATCGCCTCCTCATTT |
| *F4/80* | Forward | CATTGCGGGATTCCTACAC |
| *F4/80* | Reverse | ATTCAACCAGCAGCGATTA |
| *Cd3* | Forward | GAACCAGTGTAGAGTTGACGTG |
| *Cd3* | Reverse | CCAGGTGCTTATCATGCTTCTG |
| *Acc1* | Forward | GATGAACCATCTCCGTTGGC |
| *Acc1* | Reverse | GACCCAATTATGAATCGGGAGTG |
| *Acc2* | Forward | CGCTCACCAACAGTAAGGTGG |
| *Acc2* | Reverse | GCTTGGCAGGGAGTTCCTC |
| *Srebp1* | Forward | GGAGGCAGAGAGCAGAGATG |
| *Srebp1* | Reverse | CAGGTTCCCCATAGACAAAGAG |
| *Scd1* | Forward | GCCGTGGCTTCTTCTTCTCTC |
| *Scd1* | Reverse | GCAGTACCAGGGCACCAGCG |
| *Fasn* | Forward | TCGTCTATACCACTGCTTACTAC |
| *Fasn* | Reverse | ACACCACCTGAACCTGAG |
| *Adiponectin* | Forward | GTTGGTATCATGGTAGAGAAGAAAGCC |
| *Adiponectin* | Reverse | CCCAAGGGAACTTGTGCAGGTTGGAG |
| *Mgll* | Forward | CGGACTTCCAAGTTTTTGTCAGA |
| *Mgll* | Reverse | GCAGCCACTAGGATGGAGATG |
| *Pnpla2* | Forward | AGGACAGCTCCACCAACATC |
| *Pnpla2* | Reverse | CACATCTCTCGGAGGACCAT |
| *Lipe* | Forward | AGACACCAGCCAACGGATAC |
| *Lipe* | Reverse | GCTGGCACGGAAGAAGATAC |
| *Cpt1a* | Forward | TGTCCAAGTATCTGGCAGTC |
| *Cpt1a* | Reverse | TAGCCGTCATCAGCAACC |
| *Cpt1b* | Forward | CGAGGATTCTCTGGAACTGC |
| *Cpt1b* | Reverse | GGTCGCTTCTTCAAGGTCTG |
| *Cpt2* | Forward | CAGTCCACTTCGAGCATGCG |
| *Cpt2* | Reverse | TGGCATCAAACTTCTCCTTAGC |
| *Acadl* | Forward | CCGCCCGATGTTCTCATTC |
| *Acadl* | Reverse | CGCCATGTTTCTCTGCGATGT |
| *Acads* | Forward | TGTGCTGTGAAGTATGCC |
| *Acads* | Reverse | ATGGCCTGGTGGCTAATG |
| *Acox1* | Forward | CTCAGAGTCTGTCCAAGGCATGC |
| *Acox1* | Reverse | CTCTCATTAATCCGCATGTAGGTC |
| *Pparα* | Forward | CACGATGCTGTCCTCCTTGATG |
| *Pparα* | Reverse | CAGGCCGATCTCCACAGCAA |

# Supplementary Figures


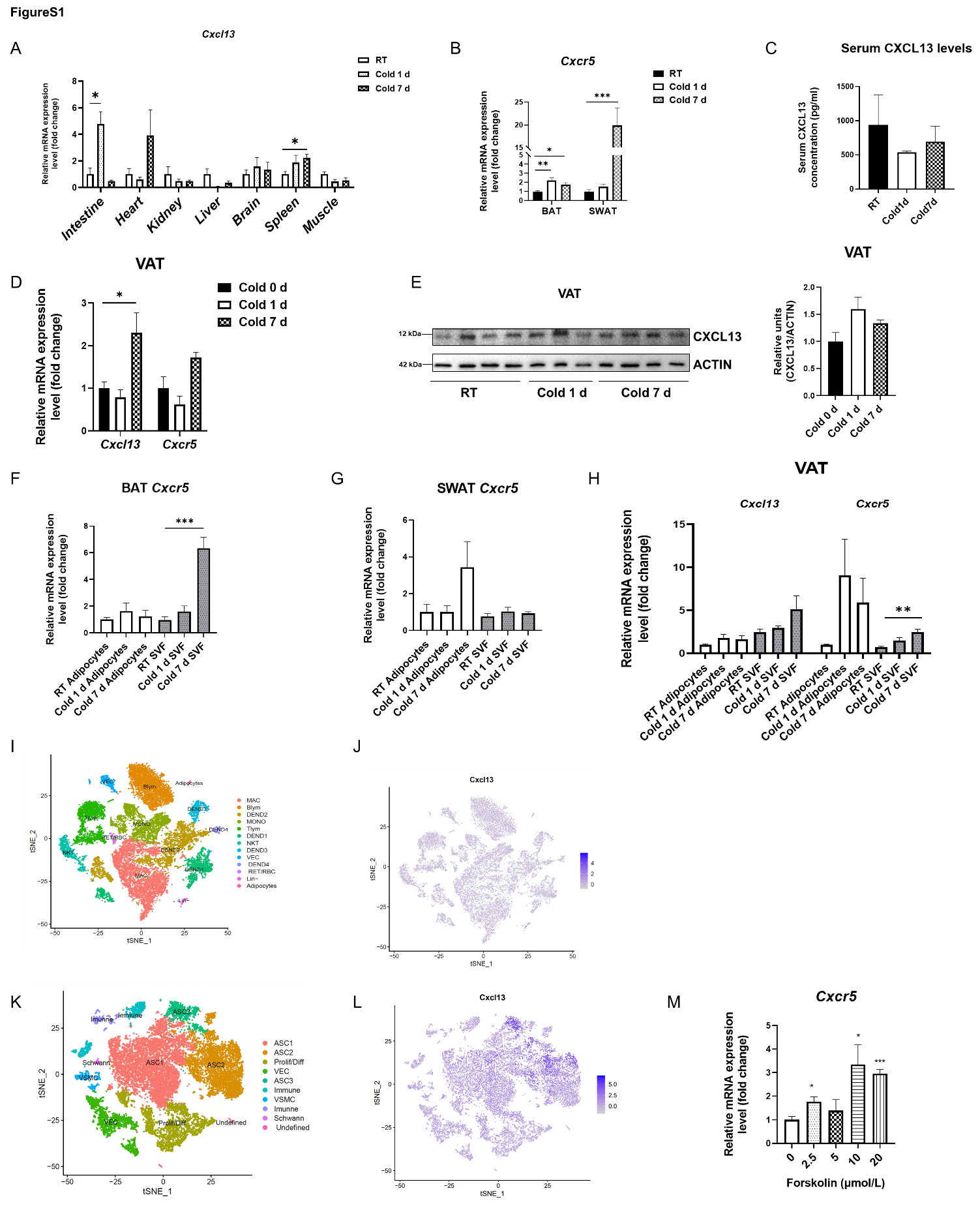


**FigureS1. Cold stimulation elevates chemokine CXCL13 expression in adipose tissue.** (A) mRNA levels of *Cxcl13* gene expression in other tissues including the intestine, heart, kidney, liver, brain, spleen, and muscle (n=3-4/group). (B) mRNA levels of *Cxcr5* gene expression in BAT and SWAT tissue (n=4-6). (C) Serum CXCL13 concentration after cold stimulation. (D) The mRNA levels of *Cxcl13* and *Cxcr5* gene expressions in the visceral adipose tissue (VAT, n=4-6/group). (E) Western blot analysis of CXCL13 protein in the VAT of the mice exposed to cold or RT (n=3-4/group, left). Quantitative measurements of CXCL13 protein to ACTIN (right). (F) Expression of *Cxcr5* relative mRNA in BAT SVF and mature adipocytes. (G) Expression of *Cxcr5* relative mRNA in SWAT SVF and mature adipocytes. (H) Expression of *Cxcl13* and *Cxcr5* mRNA levels in VAT SVF and mature adipocytes. (I) t-SNE plot of lineage marker positive (Lin+) cells. Clustering applied to the t-SNE plot identified 13 clusters, highlighted in different colors. MAC, macrophage; MONO, monocyte; DEND, dendritic cell; NKT, natural killer T-cell; Tlym, T lymphocyte; Blym, B lymphocyte; RET, reticulocyte; NEUT, neutrophil; VEC, vascular endothelial cell; Lin-, lineage-negative cells; RBC, red blood cell. (J) t-SNE plots displaying the log2 expression levels for Cxcl13 in Lin+ cells. (K) t-SNE plot of lineage marker negative (Lin-) cells Clustering identified 10 major clusters, highlighted in different colors. ASC, adipose tissue stromal cell; VEC, vascular endothelial cell; VSMC, vascular smooth muscle cells; Prolif/Diff, proliferating/differentiating cells. (L) t-SNE plots displaying the log2 expression levels for Cxcl13 in Lin- cells. (M) The mRNA expression of Cxcr5 in brown preadipocytes treated with Forskolin at different concentrations for 6 hours. Data are presented as mean ±S.E.M. *p< 0.05, **p<0.01, ***p<0.001 for all of the panels.


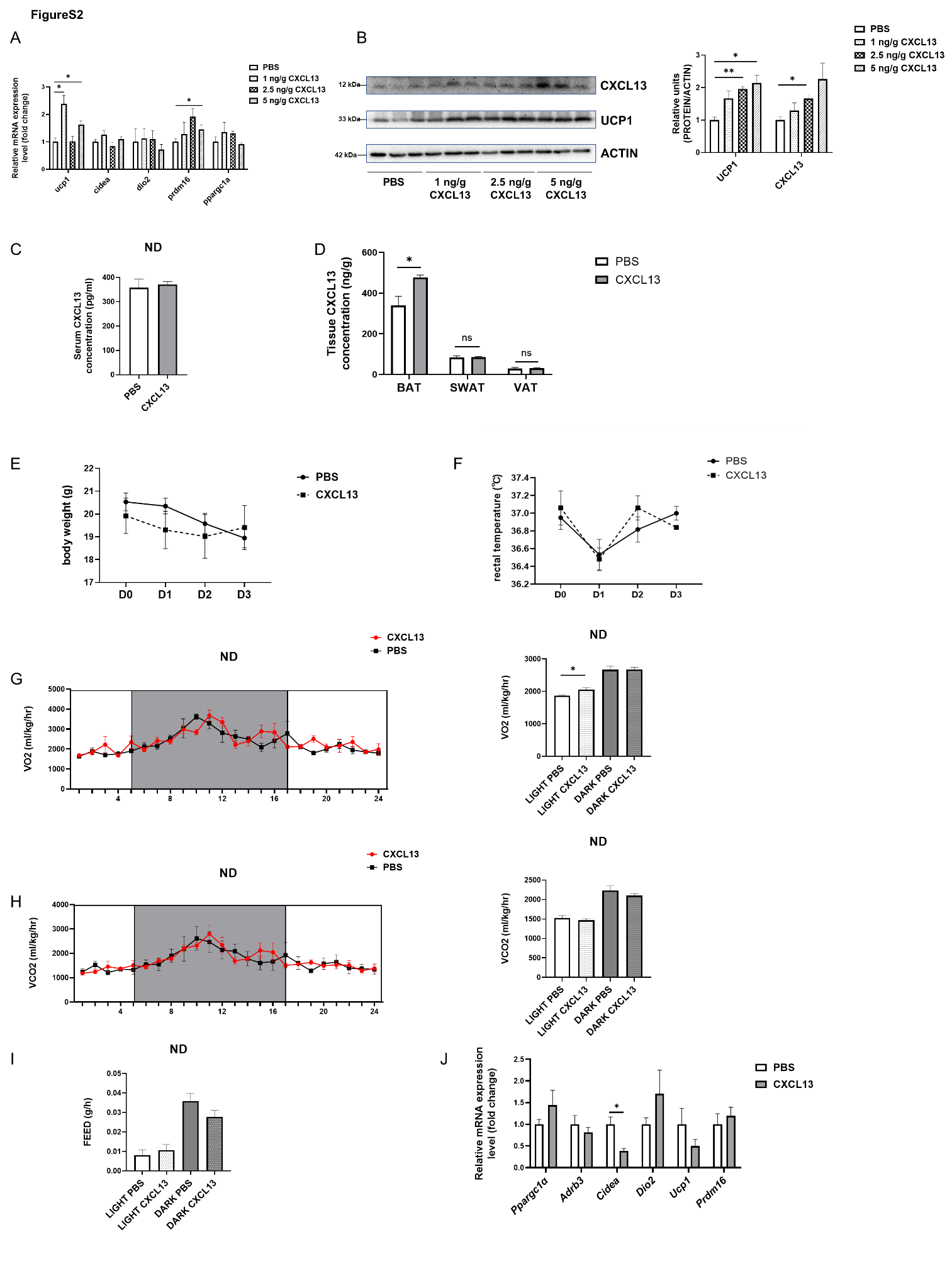


**FigureS2. Effects of CXCL13 in normal-diet mice.** (A) The mRNA levels of thermogenic genes in BAT after injection of different dosages of CXCL13 (n=4-5/group). (B) Protein levels of CXCL13 and UCP1 after injection of different dosages of CXCL13 (n=3/group). (C) Serum CXCL13 concentration after CXCL13 or PBS injection (n=5-6/group). (D) CXCL13 levels in brown adipose tissue (BAT), subcutaneous white adipose tissue (SWAT), and visceral adipose tissue (VAT) after 5ng/g/d CXCL13 injection and PBS group (n=4-5/group). (E) Body weight change during 3 days following PBS and CXCL13 injection (n=5-6/group). (F) Rectal temperature change during 3 days following PBS and CXCL13 injection (n=5-6/group). (G) Oxygen consumption (VO2) in PBS and CXCL13 injection mice. Values represent measurements of 24 hours (n = 3-4/group, left). Quantitative measurements of VO2 in these mice (right). (H) Carbon dioxide production (VCO2) in PBS and CXCL13 injection mice. Values represent measurements of 24 hours (n = 3-4/group, left). Quantitative measurements of VCO2 in these mice (right). (I) Quantitative measurements of FEED (g/h) in PBS and CXCL13 injection mice of 24 hours (n = 3-4/group). (J) mRNA levels of genes related to thermogenic function in SWAT (n=4-5/group). Data are presented as mean ±S.E.M. **p*< 0.05, ***p*<0.01 for all panels.


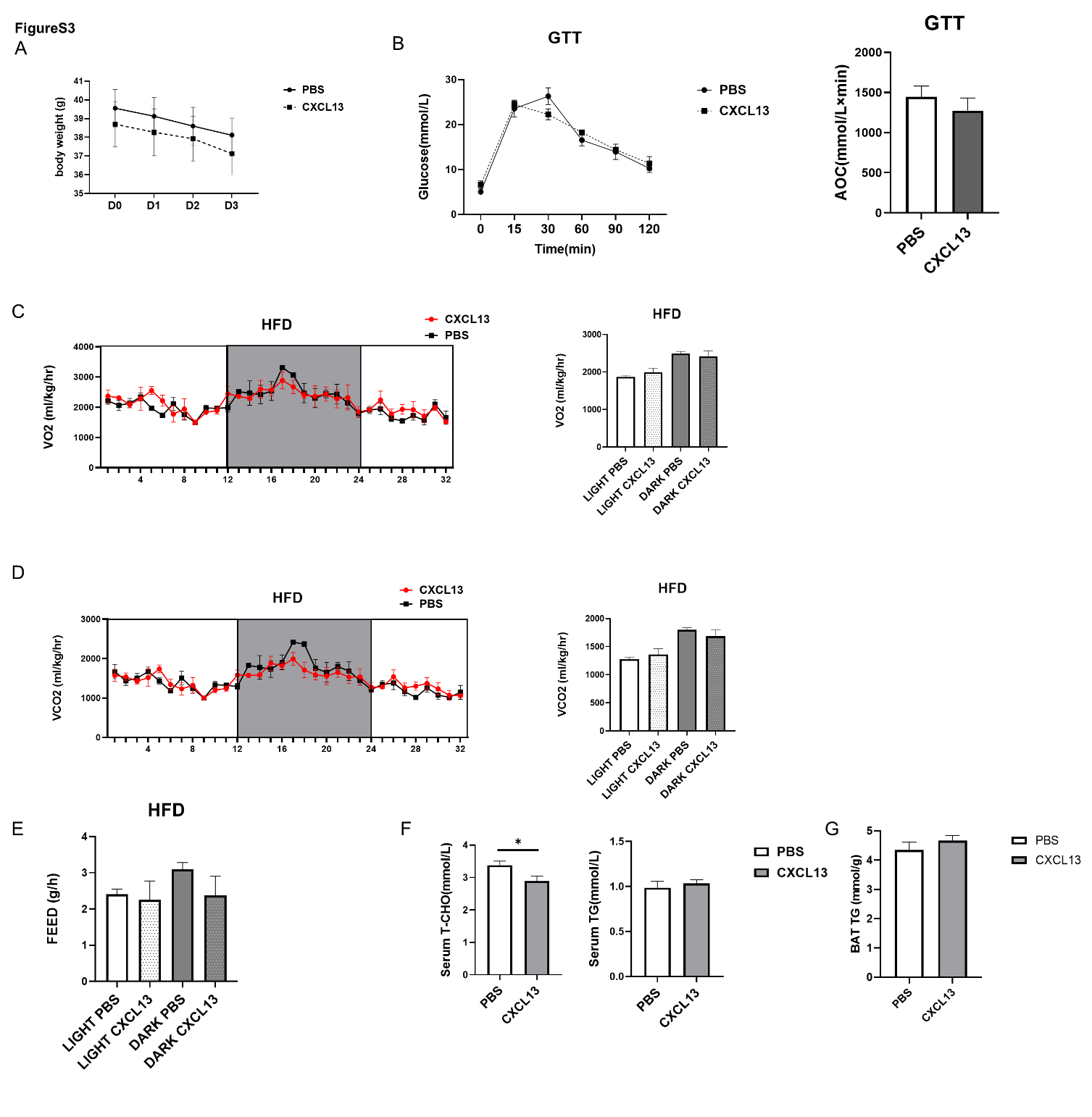


**FigureS3. Effects of CXCL13 in HFD mice.** (A) Body weight change during 3 days following PBS and CXCL13 injection (n=6-8/group). (B) glucose tolerance test (GTT, left) and the area of the curve (AOC, right) of the PBS group and CXCL13 group (n=3/group). (C) Oxygen consumption (VO2) in PBS and CXCL13 injected obese mice. Values represent measurements of 32 hours (n = 3-4/group, left). Quantitative measurements of VO2 in these mice (right). (D) Carbon dioxide production (VCO2) in PBS and CXCL13 injected obese mice. Values represent measurements of 32 hours (n = 3-4/group, left). Quantitative measurements of VCO2 in these mice (right). (E) Quantitative measurements of FEED (g/h) in PBS and CXCL13 injection mice (n = 3-4/group). (F) Serum cholesterol (T-CHO) and triglycerides (TG) levels in PBS and CXCL13 injected mice (right) after 6 hours of cold exposure (n=6-8/group). (G) BAT triglycerides (TG) levels in PBS and CXCL13 injected mice (right) after 6 hours of cold exposure (n=6-8/group). Data are presented as mean ±S.E.M. **p*< 0.05 for all panels.


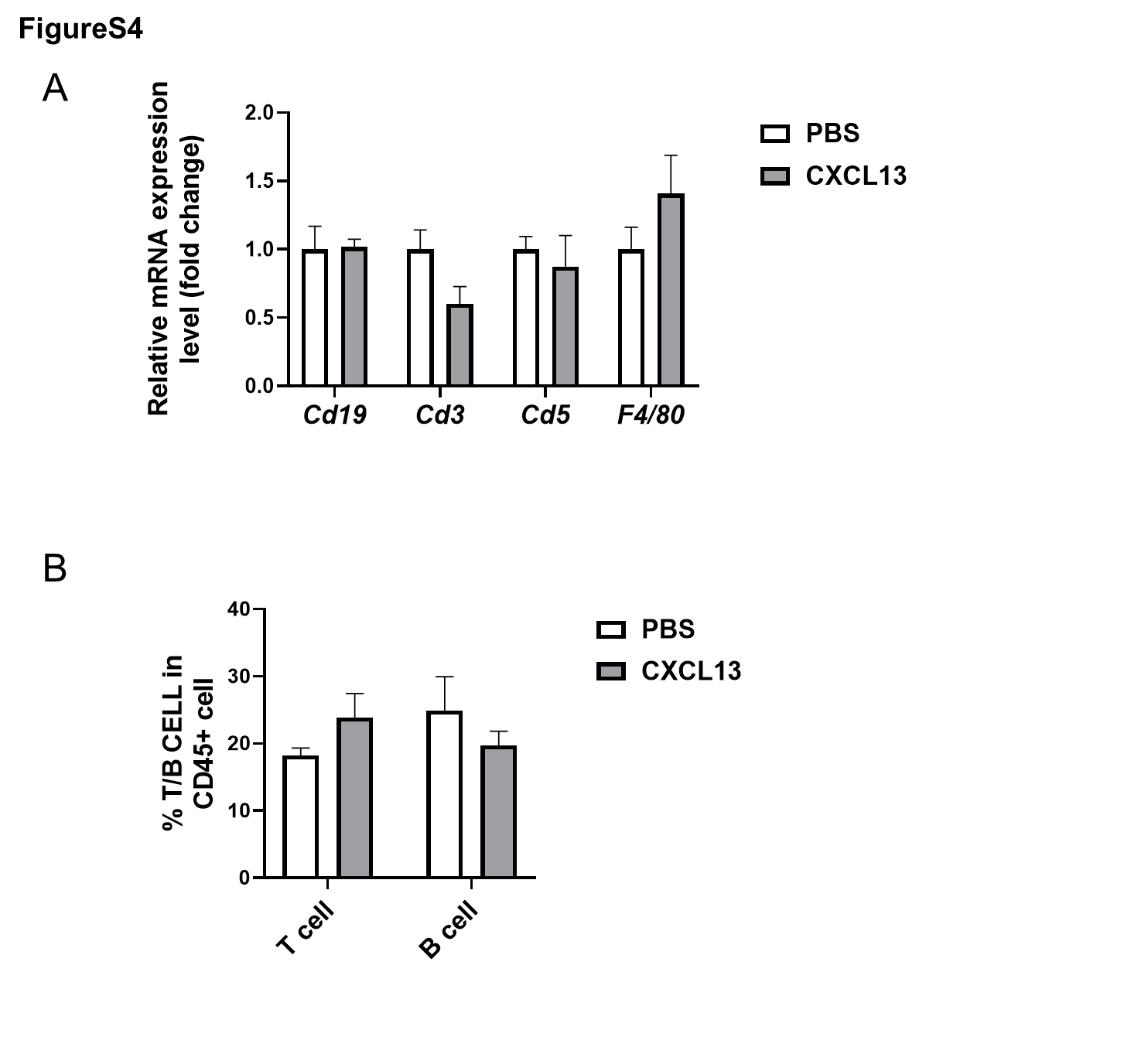


**FigureS4. Effects of CXCL13 in Macrophages in vivo.** (A) mRNA levels of immune cell-related genes in BAT in PBS and CXCL13 injection groups (n=4-5). (B) The percentage of CD3+ (T cell) and CD19+ (B cell) within the CD45+ cells population from BAT of injection mice (n=3-4). Data are presented as mean ±S.E.M.


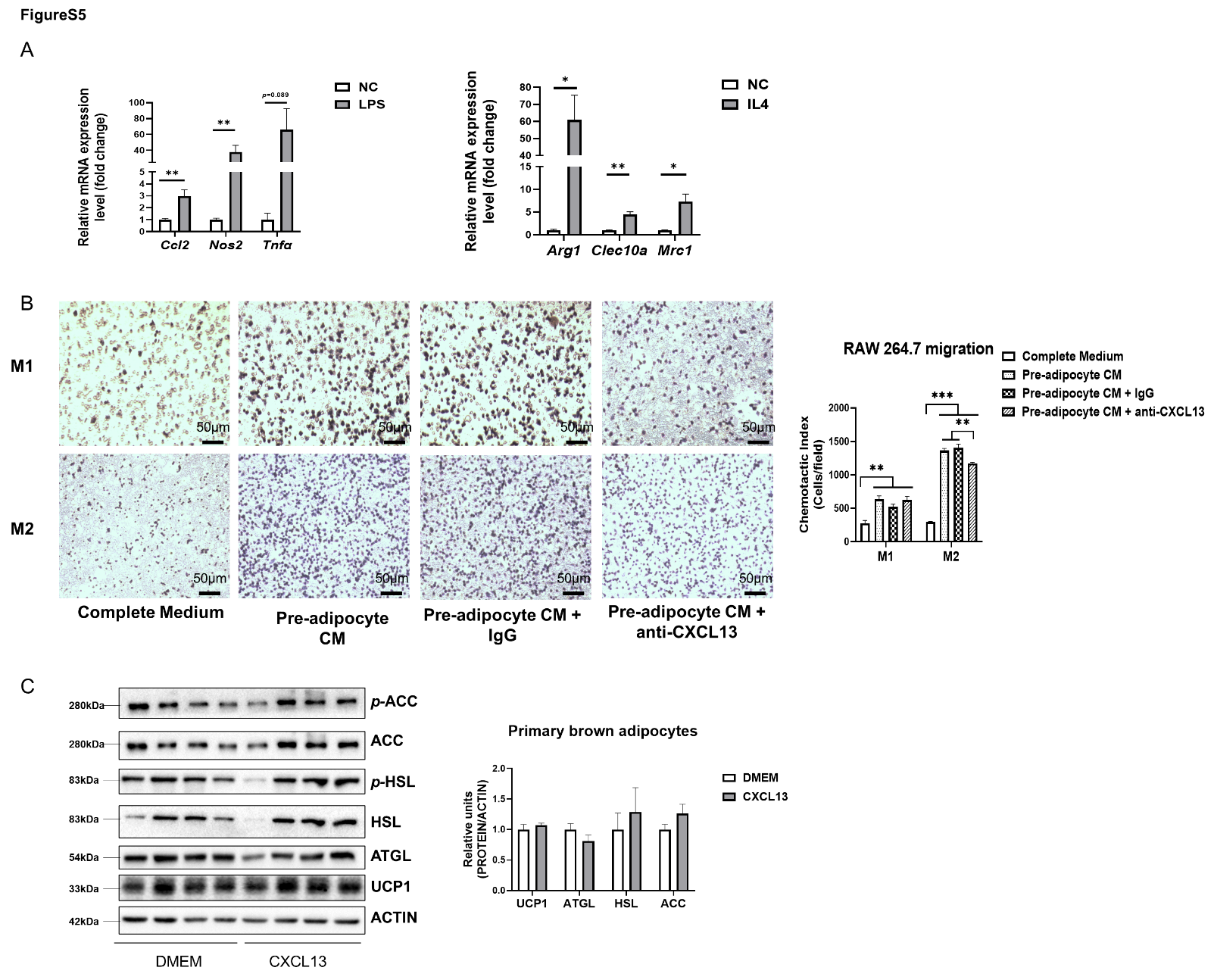


**FigureS5. Effects of CXCL13 in Macrophages in vitro.** (A) M1 BMDM activation (left, n=4-6/group) and M2 BMDM activation (right, n=4/group). (B) M1 and M2 BMDM migration assay. Macrophages were cultured with complete medium (CM), pre-adipocyte CM, pre-adipocyte CM + IgG, or pre-adipocyte CM + anti-CXCL13. Cells were fixed and stained by hematoxylin. (C) Lipogenic (ACC) and lipolysis (HSL and ATGL) and thermogenic (UCP1) protein levels in the primary brown adipocytes treated with 50 ng/mL CXCL13 (n=4/group, left), and quantitative measurements of proteins to ACTIN (right). Data are presented as mean ±S.E.M. **p*< 0.05, ***p*<0.01, ****p*<0.001 for all panels.
